# Knowledge, attitudes and practices on tuberculosis among screened immigrants in Norway. A cross-sectional study

**DOI:** 10.1016/j.jctube.2022.100326

**Published:** 2022-07-21

**Authors:** Ingunn Harstad, Andrea R. Raen, Silje Selseng, Eli Sagvik

**Affiliations:** a Department of Public Health and Nursing, Faculty of Medicine and Health Sciences, Norwegian University of Science and Technology, NO 7489 Trondheim, Norway; b Department of Pulmonary Medicine, St Olavs University Hospital, po box3250 Sluppen, N-7006 Trondheim, Norway; c Department of Infectious Disease Control, Municipality of Trondheim, Po Box 2300, Sluppen, 7004 Trondheim, Norway

**Keywords:** TB control program, Immigrant screening, KAP questionnaire, Stigma

## Abstract

**Aims:**

Most tuberculosis (TB) cases in Norway occur among immigrants from high-incidence countries. Although there is an extensive screening program for vulnerable groups, many do not appear for their screening appointment and do not understand why they require screening. This study aimed to further understand these vulnerable groups’ knowledge, attitudes and practices (KAP) regarding TB to inform the screening program and health care personnel dealing with TB.

**Methods:**

A KAP questionnaire developed by the World Health Organization (WHO) and adjusted to Norwegian conditions was used. The study has a cross-sectional design. One study group was immigrant students receiving primary screening in the municipality (MVIC) who completed an English questionnaire; the other was immigrants who were referred to hospital (POPD) for follow-up of screening results. They were interviewed with a translator when necessary. Statistical analyses to describe and compare groups of participants were done.

**Results:**

Altogether, 275 persons were eligible, and 219 (85%) participated. In the MVIC group 184 persons (86%) participated and in the POPD group 35 persons (80%). The mean knowledge score was 5.53 (maximum score: 11) with no significant differences between study groups or associations with demographics or length of education. There were serious knowledge gaps related to TB symptoms and transmission. Approximately half would have reacted with fear or surprise if they had TB, and 60% were afraid of being infected. Only 14% would avoid a person with TB.

**Conclusion:**

The mean knowledge score was reasonably good but with some serious knowledge gaps. We detected fear of being infected or having TB disease but no serious stigma. More information and teaching about TB catered towards different immigrant groups are necessary.

## 1 Background

### 1.1 General background

Tuberculosis (TB) is an infectious disease caused by *Mycobacterium tuberculosis*. An estimated 23 % of the world's population is infected and approximately 5–10 % of the infected will develop the disease during their lifetime [1]. Those who are infected but not ill have latent TB. By giving prophylactic treatment to those with latent TB, the risk for developing TB can be reduced.

In 2017 the World Health Organization (WHO) estimated 10.4 million new TB cases and 1.7 million deaths from TB worldwide [1]. During recent decades the WHO has created goals and strategies for eliminating TB, and the most recently to End TB by 2030 [2]. When a low-incidence country is approaching the elimination phase (0.2–1 TB cases per 100 000/year), more extensive treatment of latent TB is advised to further reduce the number of new cases [3].

### 1.2 TB epidemiology and screening in Norway

In Norway 268 new TB cases were registered in 2016 and approximately 90 % of these occurred in immigrants [4]. Norway is approaching the elimination phase of TB. However, to reach the goal of TB elimination, further efforts are required. There is an extensive compulsory screening program for immigrants from high incidence countries, defined as > 40 cases per 100 000 inhabitants per year, and for all asylum seekers (applied for asylum but not yet granted) and refugees (granted asylum either by United Nations or after application in Norway). Further: the whole group will be called immigrants. The aim is both to improve each immigrant’s prognosis and to stop transmission by detecting cases of active TB earlier and treating cases of latent TB. Previous structural studies on the screening program have shown challenges in the follow-up of initial screening findings; In a study on screening asylum seekers, approximately-one third of patients with a positive X-ray finding were not followed up, and only one-third with a positive Tuberculin skin test had a follow-up [5]. One of the problems detected in another study is that the immigrants did not appear for their appointment for follow-up at the pulmonary out-patient department (POPD) [6]. We can speculate that reasons for this failure to appear include; not knowing why they have an appointment, not understanding the reasons for screening, or practical obstacles. However, we do not know much about these questions.

### 1.3 KAP studies on TB

The WHO has developed a questionnaire template to investigate knowledge, attitudes and practices (KAP) regarding TB [7]. This tool can be used among health care personnel, different patient groups, and the general population. Some KAP studies among specific immigrant groups in other low-incidence countries e.g. Great Britain, the US and Sweden, show a diversity of results; however, they all show that more knowledge is necessary [8], [9], [10]. Each study has estimated a good knowledge score from the total possible score in that specific study and no general cut-off has been established. Specifically, not much is known about immigrant's KAP towards TB in Norway, and no such studies have previously been conducted.

### 1.4 Aims

This study aimed to further understand knowledge, attitudes and practices regarding TB of immigrants from high incidence countries. By improving our understanding of the immigrants KAP regarding TB, health care workers and screening program planners can be better informed on this diverse group. This could possibly lead to e.g. better targeted health information and screening routines.

## 2 Methods

In this descriptive, cross-sectional study, the WHO‘s KAP template [7] was used to develop a questionnaire with 22 questions on sociodemographic background information, KAP on TB and questions related to the actual screening setting.

### 2.1 Study populations

Two study groups, both from high incidence TB countries [11] and included in the ordinary national TB screening program, participated. All participants were 18 years or older and both genders were represented.

Group one included patients who were referred to the POPD at St. Olavs University Hospital because of positive findings on TB arrival screening: either a positive IGRA test, findings on chest X-ray or information about previously being treated for TB. This group included students, asylum seekers, refugees and others.

Group two included students who planned to stay in Norway for a shorter or longer period. They were all invited to their primary screening at the municipality’s office for vaccination and infection control (MVIC).

### 2.2 The questionnaire

The WHO‘s template for KAP questionnaires on TB (7) was used to develop the questionnaire. It was modified for immigrants in Norway and assessed sociodemographic information, previous TB treatment, and whether they knew anybody with TB. It also included nine questions on knowledge on TB, four questions on attitude, and practical questions related to their appointment. The questionnaire was created in Norwegian and translated to English by a bilingual medical doctor.

### 2.3 Data collection

Two medical students collected the data as part of their student thesis. During the study period all screening patients were asked to participate at both study sites.

Group one underwent an ordinary appointment at the POPD. They received an ordinary appointment letter including brief information in their own language. Upon arrival, they were asked by the medical students if they wanted to participate in the study and received time to think before providing an answer. If they were affirmative, they were interviewed by the medical students with a translator when necessary, and the medical students recorded their replies. The interviews were performed before consultation with the physician. Data collection was conducted from 05.02 to 11.04.2018 and again from 21.08 to 09.10. 2018. The number of patients with appointment who arrived, how many translators were booked and how many agreed to participate in the study were recorded.

Group two received an e-mail with information about the screening; in this e-mail, they were also informed about the study. When they came to the MVIC, they were asked by either the students or local health care personnel if they wanted to participate. If they were affirmative, they completed the KAP questionnaire in English by themselves before they underwent their appointment and examination. The data collection was performed 13.09–14.10.2017 and 24.01.2018. The number of students with an appointment and the number who participated in the study was recorded, but not how many arrived the actual days.

### 2.4 Statistical analysis

The data were manually entered into SPSS Statistic Software version 25 by the two medical students. Random cross-checking was performed. Some questions required one answer and others allowed multiple responses. If respondents gave multiple responses on questions requiring only one answer, these cases were coded as missing data.

A knowledge-score from 0 to 11 was calculated from the nine knowledge questions. This score was normally distributed and the mean and standard deviation (SD) were used for analysis. A *t*-test was used to compare the knowledge-score between two groups, and an ANOVA was performed for multiple groups. To analyze whether knowledge score varied with answers on attitude questions, an ANOVA test was used and Chi square test was used as an alternative to ANOVA. The independent variables were: education, country of origin, gender, age, time in Norway, and personal experience with TB. The dependent variables were: knowledge score and attitude towards TB. P < 0.05 was regarded as statistically significant.

### 2.5 Ethics

The Regional Committee for Medical Research Ethics approved this study after some minor adjustments of the protocol (2017/904). Each participant was asked for informed consent and consented by participation.

## 3 Results

### 3.1 Description of the study population

Altogether 257 persons were eligible for study participation and 219 (85 %) participated. The MVIC group included 213 eligible persons, of whom 184 (86 %) participated. The POPD group included 44 eligible persons, of whom 35 (80 %) participated (Table 1). Altogether 134 (61 %) males and 85 females participated. In the POPD group 7 (20 %) persons were < 20 years old and 18 (51 %) were > 30 years old. In the MVIC group 167 (91 %) persons were between 20 and 30 years old. Most persons in the MVC group stayed for education: n = 181 (98 %) whereas the POPD group stayed for a mixture of reasons; education, work, family reunion and refuge reasons (Table 1). In the MVIC group 124 (67 %) persons had stayed for <2 months, 173 (96 %) had >13 years of completed education and 135 (73 %) were from Asia (Table 1).

**Table 1:** Study participants by sociedmographic status, study site and total numbers.

**Study location**	**MVIC (%)**	**POPD (%)**	**Total numbers (%)**
Invited	213	44	257
Participants (% of invited)	184 (86)	35 (80)	219 (85)
**Gender**			
Male	114 (62)	20 (57)	134 (61)
Female	70 (38)	15 (43)	85 (39)
**Age groups**			
<20	0	7 (20)	7 (3)
20–25	119 (65)	2 (6)	121 (55)
26–30	48 (26)	8 (23)	56 (26)
>30	17 (9)	18 (51)	35 (16)
**Reason for stay¤**			
Education	181 (98)	4 (12)	185 (85)
Work	2 (1)	8 (24)	10 (5)
Family reunion	1 (1)	10 (30)	11 (5)
Refugee	0	10 (30)	10 (4)
Other	0	1 (1)	1 (1)
**Time in Norway #**			
<2months	124 (67)	0	124 (57)
2–12 months	38 (21)	18 (51)	56 (26)
>12 months	18 (10)	17 (49)	35 (16)
**Completed education**			
No school/<6 years	0	4 (11)	4 (2)
6–10 years	1 (1)	3 (9)	4 (2)
11–13 years	6 (3)	13 (37)	19 (9)
>13 years	173 (96)	15 (43)	188 (86)
**Continent of origin**			
Europe	15 (8)	5 (14)	20 (9)
Asia	135 (73)	16 (49)	151 (69)
Africa	27 (15)	12 (34)	39 (18)
South America	7 (4)	1 (3)	8 (4)

POPD: pulmonary Out-Patient Department, MVIC: Municipal office for Vaccination and Infection Control. ¤missing data on 2 cases, ¤missing data on 2 cases, #missing data on 4 cases.

In total 11 (5 %) patients had previously been treated for TB, including 4 from the MVIC and 7 from the POPD. Thirty (14 %) participants knew somebody with TB, including 19 from the MVIC and 11 from the POPD.

### 3.2 Knowledge

**Fig. 1:**
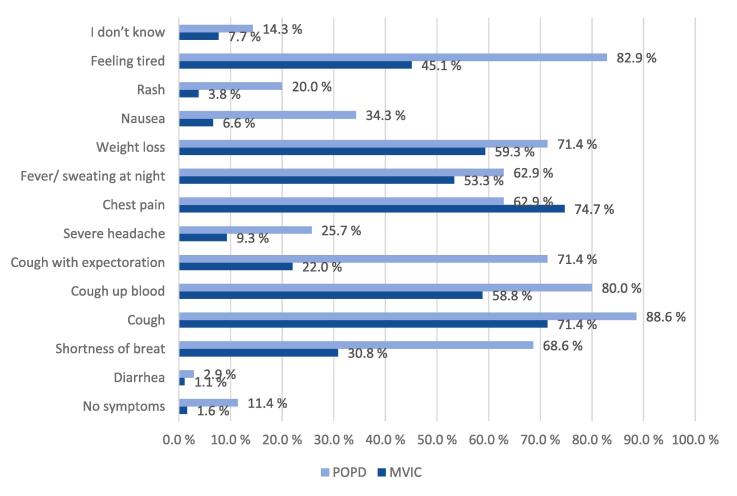
What are the signs and symptoms of lung tuberculosis?

**Fig. 2:**
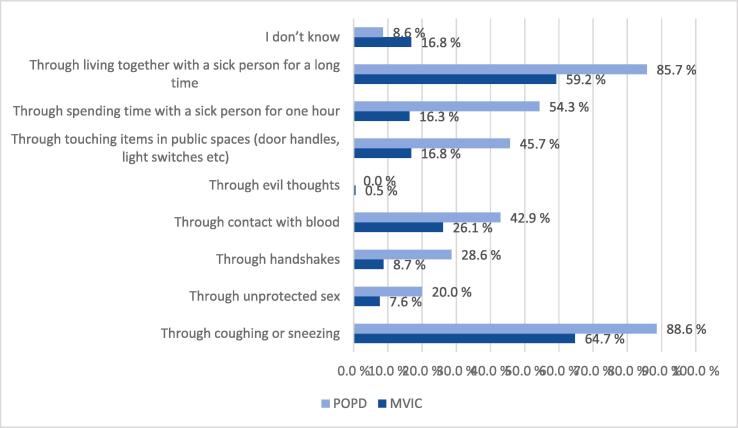
How can a person get tuberculosis?

TB was viewed as a serious or very serious disease among 85 % of the study participants. Forty percent of the participants agreed wrongly that all infected persons will become ill. More than half of the participants at both study sites could correctly identify the three most important symptoms of TB as fever, cough and weight loss (Fig. 1). The question about infection transmission was correctly answered as coughing and sneezing by 119 (64.7 %) persons at the MVIC and 31 (88.6 %) at the POPD, and as living with an infectious person for a long time by 109 (59.2 %) persons at the MVIC and 30 (85.7 %) at the POPD. However, a high proportion believed wrongly that TB could be transmitted through blood (Fig. 2), and several also believed in transmission by touching surfaces and through unprotected sex (Fig. 2).

Among the participants 180 (82 %) subjects believed TB can be treated but disagreed about getting well without treatment. Altogether 127 (58 %) subjects did not know that there is free treatment for TB in Norway.

A mean knowledge score was 5.53 (95 % CI 5.26–5.80) of 11 for all study participants; there were no significant differences between study sites, gender, age groups, time in Norway, reasons for staying, continent of origin, previous treatment for TB or knowing somebody with TB (Table 2). The mean knowledge score was 4.89 for those with <13 years of education and 5.60 for those with >13 years of education (p = 0,09).

**Table 2:** Mean knowledge score by sociodemographic status, study location and TB experience.

	**N (%)**	**Mean knowledge score**	**SD**	**95 % CI**	**p-value**	
**Participants**	219	5.53	2.04	5.26–5.80	*t*-test	**ANOVA**
**Study site**						
POPD	35 (16)	5.20	1.83	4.89–5.51	0.297	
MVIC	184 (84)	5.59	2.08	5.44–5.75		
**Gender**						
Male	134 (61)	5.48	2.04	5.13–5.83	0.632	
Female	85 (39)	5.62	2.05	5.16–6.06		
**Age groups**						
<=25	128 (58)	5.46	2.09	5.28–5.65	0.576	
>=26	91 (42)	5.62	1.99	5.41–5.83		
**Time in Norway #**						
<2months	124 (57)	5.47	2.07	5.28–5.65	0.497	
>=2months	91(42)	5.66	2.00	5.45–5.87		
**Reason for stay**						
Education	185 (84.5)	5.62	2.10	5.32– 5.93		0.51
Work	10 (4.5)	5.30	1.34	4.34- 6.26		
Family reunion	11 (5.1)	4.73	2.15	3.28- 6.17		
Refugee	10 (4.5)	5.30	1.06	4.54- 6.06		
**Completed education**						
<=13 years	27	4.89	2.01	4.10–5.68	0.09	
>13 years	188	5.60	2.04	5.31–5.90		
**Continent of origin**						
Europe	20 (9)	6.00	2.34	4.91–7.10		0.113
Asia	152 (69)	5.33	2.061			
Africa	39 (18)	5.795	1.69	5.25–6.34		
South America	8 (4)	6.75	2.05	5.03–8.47		
**Previously treated for TB ¤**						
Yes	11 (5)	5.64	1.75	4.46–6.81	0.906	
No	198 (90)	5.56	2.08	5.27–5.85		
**Know somebody with TB §**						
Yes	30 (14)	5.93	2.08	5.16–6.71	0.219	
No	168 (77)	5.44	2.00	5.14–5.75		

POPD: Pulmonary Out-Patient Department, MVIC: Municipal office for Vaccination and Infection Not Control, #missing data on 4 cases from MVIC, ¤: 8 don‘t know, § 19 don‘t know, SD: Standard deviation, altogether 2 missing cases for knowledge score.

### 3.3 Attitude

When asked about fear of becoming infected, 130 (59 %) persons were fearful. Fear 54 (25 %) and surprise 62 (28 %) were the most common reactions regarding being diagnosed with TB. When asked about feelings towards a TB patient, 38 (17 %) would avoid the patient, and 129 (59 %) would behave as normally. Attitudes in their home country differed slightly; 61 (28 %) would be friendly but avoid the patient, 71 (32 %) would help and support the patient; only 7 (3 %) persons replied that they would mostly avoid the patients.

There were no association between knowledge score and fear of being infected, reaction towards one's own diagnosis, reaction towards others‘ diagnosis, why they had the appointment or having learned anything about TB after arriving in Norway.

When asked about whether they had learned anything about TB after arriving in Norway, 160 (73 %) persons answered yes; 143 (78 %) in the MVIC group and 17 (49 %) in the POPD group. In the MVIC group 92 (62 %) had learned from the information letter provided with the appointment letter for their TB check-up. Other possibilities included friends, doctors, the internet and public information sites. There was an association between having learned anything about TB after arrival in Norway with study site (p < 0.001) and years of education (p = 0.001) (Table 3).

**Table 3:** Have learnt about TB after arrival in Norway versus time in Norway, study location and years of school.

	Learnt: yes	Learnt: no	sum	Chi-square p
**Time in Norway**				
< 2 months	96	27	123	0.067
>= 2 months	60	31	91	
**Study site**				
MVIC	143	40	183	<0.001
POPD	17	18	35	
**Years of school**				
<=13 years	11	16	27	0.001
>13 years	146	41	187	

POPD: Pulmonary Out-patient department, MVIC: Municipal office for Vaccination and Infection. Not all numbers are adding up due to missing values.

There were no associations between “have had TB or know somebody with TB” and how serious the immigrants think TB is, if they think there is any effective treatment, and if they fear being infected.

### 3.4 About the appointment

In total 33 (15 %) persons had subjective problems keeping their appointment, including 28 (15 %) in the MVIC group and 5 (14 %) in the POPD group. Additionally, 92 (42 %) persons rightly believed their appointment was related to TB examinations, including 73 (40 %) in the MVIC group and 19 (54 %) in the POPD group.

## 4 Discussion

In this study of KAP among immigrants from high incidence countries screened for TB, the mean knowledge score was 5.53 out of 11. The participants knew about the most important symptoms of TB and agreed that TB is a serious disease. However, there were serious knowledge gaps on disease transmission: like for example believing TB can be transmitted through blood, through unprotected sex or by touching surfaces, and not knowing that living with an infectious person for a long time can transmit TB. There were no significant difference in knowledge score between the two study sites, nor between years of education (p-value: 0.09). Approximately 70 % of the immigrants had learned about TB after arrival in Norway: this was associated with study site and years of education.

Fear or surprise was the most common feelings towards being diagnosed with TB, however, only 14 % would have avoided a patient with TB. More than half of the participants did not know their appointment was related to TB examinations.

### 4.1 Knowledge

The general knowledge score was 5.53 out of 11. This agrees with or is slightly better than a similar study among language students in Sweden, which reported a score of 2.7 out of 8 [8]. A study from the USA among foreign-born and US-born patients with latent TB reported a score of 6.86 and 7.21 out of 11, respectively [12]. A Swedish study and a study from the Philippines [13] reported positive associations between knowledge scores and >12 years of education. There were no significant associations between knowledge score and demographics in our study, but a study from India identified an association between being illiterate, female and low knowledge score.

Most immigrants knew the most important signs and symptoms of TB, similar to other studies. However, misconceptions about disease transmission and factors increasing disease risk were serious but similar to previous studies from the US and Sweden [8], [9], [12]. This could lead to unnecessary fear of infection but also unawareness of important risks for transmission. Most believed treatment to be effective and necessary for healing but several replied that they did not know, or it was unnecessary; this is important from a health education point of view.

Approximately 70 % had learned about TB after arrival, which was associated with study site (MVIC) and years of education. In a Swedish study, <30 % of participants had learned about TB after arrival [8]. The letter about the appointment and information from the university were important sources of information for Norwegian students. In an Indian study on KAP before and after providing community-based health information on TB, knowledge increased after intervention [14]. Information should be part of the routine in order to have the desired effect.

### 4.2 Attitude

We wanted to investigate whether stigma or negative attitudes towards TB patients were prevalent among immigrants screened for TB, but we did not find it feasible to develop an attitude score. Fear or surprise were the most common feelings towards being diagnosed with TB. A qualitative study among African men with TB in London reported feeling shocked, surprised and bewildered when diagnosed [15]. In our study only 14 % would avoid a patient with TB. When asked about how TB patients are treated in their home country, the results were slightly more negative: 33 % would support and help whereas 30 % would be friendly but attempt to avoid the patient. In the Swedish study, many subjects were afraid of TB, but as opposed to our study, most responders wanted to avoid TB patients and altogether had a low attitude score [8]. In a qualitative US study, the participants did not want to share their TB status with others but mostly experienced support when they did, with some exceptions [9]. However, in another qualitative study among TB patients in London, there was a strong feeling of stigma but actual experience of stigma mainly occurred among those who had experienced isolation as an infection control measure [16].

### 4.3 Appointment

Approximately 95 % received their appointment letter, and only 15 % responded that it was difficult to attend their appointment. Nevertheless, many of those who declared there was no problem, required extra phone calls or help finding the clinic. We do not have any information from the most interesting group, which was those who did not appear for their appointment. A previous study from our POPD showed that by intensifying the information provided to patients, giving them an appointment shortly after primary screening, calling them and simplifying the examinations on arrival, more patients keep their appointment [6].

Altogether more than half did not know why they had their appointment, but in the POPD group it was slightly better. They had all seen a public health nurse and been examined for TB before this referral to POPD. In the Swedish study, most students had also been in touch with health care related to screening procedures and still their knowledge score was quite low [8]. It seems each meeting with the health care must be used for relevant, personalized information and teaching about TB.

### 4.4 Strengths and limitations

KAP is a well-established and tested questionnaire used assessing health care personnel and patients in different settings. We adjusted the questionnaire to a Norwegian setting and had it translated to English by a bilingual medical doctor making it a robust test tool. However, our results can not be directly compared to other studies and some of the questions are not previously tested.

Slightly different methods were used in our two study groups. Students in the MVIC group completed the questionnaire by themselves without anyone to correct misunderstandings. For one question in particular; 31 replies were excluded because they included more than one response. Only 3 complete questionnaires were excluded. The participants completed their questionnaire completely anonymously and without any personal support. Thus, we do not know whether they used any aids, e.g. the internet, which could have improved the knowledge score.

In the POPD group, the questionnaire was read to the participant either by a translator or medical student, both of whom were present. We have no control of what the translator said, and the participants could not reply anonymously. Thus, there were less misunderstandings, but the participant might not feel free to give the answer she or he wanted.

We do not know the exact number of students who arrived for tests on the day of the study, but we do know how many had an appointment. Up to 85 % of those with an appointment participated, which make this sample representative for immigrant students that year. However, another year the students might have a different background and reply differently to a KAP questionnaire.

In the POPD group, 35 (80 %) patients participated, which is acceptable from a representative point of view. The total numbers were lower than planned because fewer asylum seekers arrived during this period, and new screening guideline included less immigrants than before. Compared to all referred to POPD for TB in the study period, fewer of the study participants required translator and more spoke English, maybe they also had increased knowledge? The background of immigrants with positive findings on arrival screening are diverse and frequently changing. That must be taken into account when these KAP results are interpreted. Only those who appeared could be asked about problems with the appointment, which limits the usefulness of this question.

## 5 Conclusions

The general knowledge score was reasonably good in this KAP study among immigrants screened for TB. However, important knowledge gaps on symptoms and transmission tell us that health information on TB among vulnerable groups must be intensified to ensure that patients with symptoms suspect of TB receive proper and timely health care to avoid treatment delay and onward transmission, and prevent unnecessary stigma and avoidance of patients. Additionally, well-educated persons responded well to the health information in the appointment letter/mail. Therefore, health education must be tailored to the needs of different immigrant groups and each meeting with health care personnel must be used for relevant, personalized teaching about TB.

## Declaration of Competing Interest

The authors declare that they have no known competing financial interests or personal relationships that could have appeared to influence the work reported in this paper.
